# SHARPIN Is Essential for Cytokine Production, NF-κB Signaling, and Induction of Th1 Differentiation by Dendritic Cells

**DOI:** 10.1371/journal.pone.0031809

**Published:** 2012-02-14

**Authors:** Zhe Wang, Anna Sokolovska, Rosemarie Seymour, John P. Sundberg, Harm HogenEsch

**Affiliations:** 1 Department of Comparative Pathobiology, Purdue University, West Lafayette, Indiana, United States of America; 2 The Jackson Laboratory, Bar Harbor, Maine, United States of America; South Texas Veterans Health Care System and University Health Science Center San Antonio, United States of America

## Abstract

Spontaneous mutations of the *Sharpin* (*SHANK-associated RH domain-interacting protein*, other aliases: *Rbckl1*, *Sipl1*) gene in mice result in systemic inflammation that is characterized by chronic proliferative dermatitis and dysregulated secretion of T helper1 (Th1) and Th2 cytokines. The cellular and molecular mechanisms underlying this inflammatory phenotype remain elusive. Dendritic cells may contribute to the initiation and progression of the phenotype of SHARPIN-deficient mice because of their pivotal role in innate and adaptive immunity. Here we show by flow cytometry that SHARPIN- deficiency did not alter the distribution of different DC subtypes in the spleen. In response to TOLL-like receptor (TLR) agonists LPS and poly I:C, cultured bone marrow-derived dendritic cells (BMDC) from WT and mutant mice exhibited similar increases in expression of co-stimulatory molecules CD40, CD80, and CD86. However, stimulated SHARPIN-deficient BMDC had reduced transcription and secretion of pro-inflammatory mediators IL6, IL12P70, GMCSF, and nitric oxide. Mutant BMDC had defective activation of NF-κB signaling, whereas the MAPK1/3 (ERK1/2) and MAPK11/12/13/14 (p38 MAP kinase isoforms) and TBK1 signaling pathways were intact. A mixed lymphocyte reaction showed that mutant BMDC only induced a weak Th1 immune response but stimulated increased Th2 cytokine production from allogeneic naïve CD4^+^ T cells. In conclusion, loss of *Sharpin* in mice significantly affects the immune function of DC and this may partially account for the systemic inflammation and Th2-biased immune response.

## Introduction

SHARPIN was originally identified in post-synaptic densities of excitatory synapses in the brain of rats [1], but this protein is widely expressed in a variety of tissues [2]. Two allelic, autosomal recessive mutations in the *Sharpin* gene occurred spontaneously in two inbred strains of mice, C57B/KaLawRij-*Sharpin^cpdm^/Sharpin^cpdm^* and CBy.Ocb3/Dem-*Sharpin^cpdm-Dem^*/*Sharpin^cpdm-Dem^*, resulting in premature termination of mRNA synthesis and absence of a functional protein product [2]. Despite different genetic backgrounds, both mutations cause similar inflammatory disease with severe chronic progressive dermatitis and defective development of secondary lymphoid organs [2]–[4]. The dermatitis becomes clinically apparent at about four weeks of age. There are accumulations of eosinophils, neutrophils and macrophages in the skin of *Sharpin^cpdm^/Sharpin^cpdm^* mutant mice (hereafter referred to as *cpdm* mice) associated with increased expression of Th2 cytokines in the skin and in the supernatants of activated splenocytes [5]. The mice have an impaired delayed type hypersensitivity response and decreased secretion of IFNγ [5], indicating a defect in Th1 immune responses and a bias towards a Th2 immune response. Systemic treatment of *cpdm* mice with recombinant IL12 caused complete remission of the dermatitis [5]. Neutralization of IL5 by antibody treatment or crosses with IL5-deficient mice reduced the number of circulating and cutaneous eosinophils, but failed to reduce the onset and severity of the dermatitis [6].

Recently, three independent groups identified SHARPIN as an essential component of the linear ubiquitin chain assembly complex (LUBAC) that regulates TNFα-induced canonical NF-κB signaling [7]–[9]. SHARPIN-deficient mouse embryonic fibroblast (MEF) were sensitized to TNFα-induced apoptosis and cell death was implicated as a factor in the dermatitis of *cpdm* mice [7]–[9].

Dendritic cells (DC) have a sentinel role in sensing pathogen or danger signals and initiate and direct activation of the adaptive immune response [10]. Activated and mature DC can carry processed antigenic peptides, migrate to lymphoid organs, and induce T-cell-mediated immune responses or tolerance. DC direct the differentiation of CD4^+^ T cells, and hence the type of immune response, through the selective secretion of cytokines. We hypothesized that defective cytokine secretion by DC contributed to the Th2-biased inflammatory phenotype in SHARPIN-deficient mice. The studies reported here found that lack of SHARPIN protein in BMDC caused defective expression of pro-inflammatory mediators and impaired NF-κB activation upon ligand stimulation. The ability of *cpdm* BMDC to stimulate Th1 cytokine production in allogeneic CD4^+^ T cells was compromised. Taken together, these results reveal that SHARPIN is a novel regulatory molecule in DC biology and suggest that the dysregulated function of SHARPIN-deficient DC plays a role in the *cpdm* phenotype.

## Results

### Characterization of *Sharpin*

**Figure 1:**
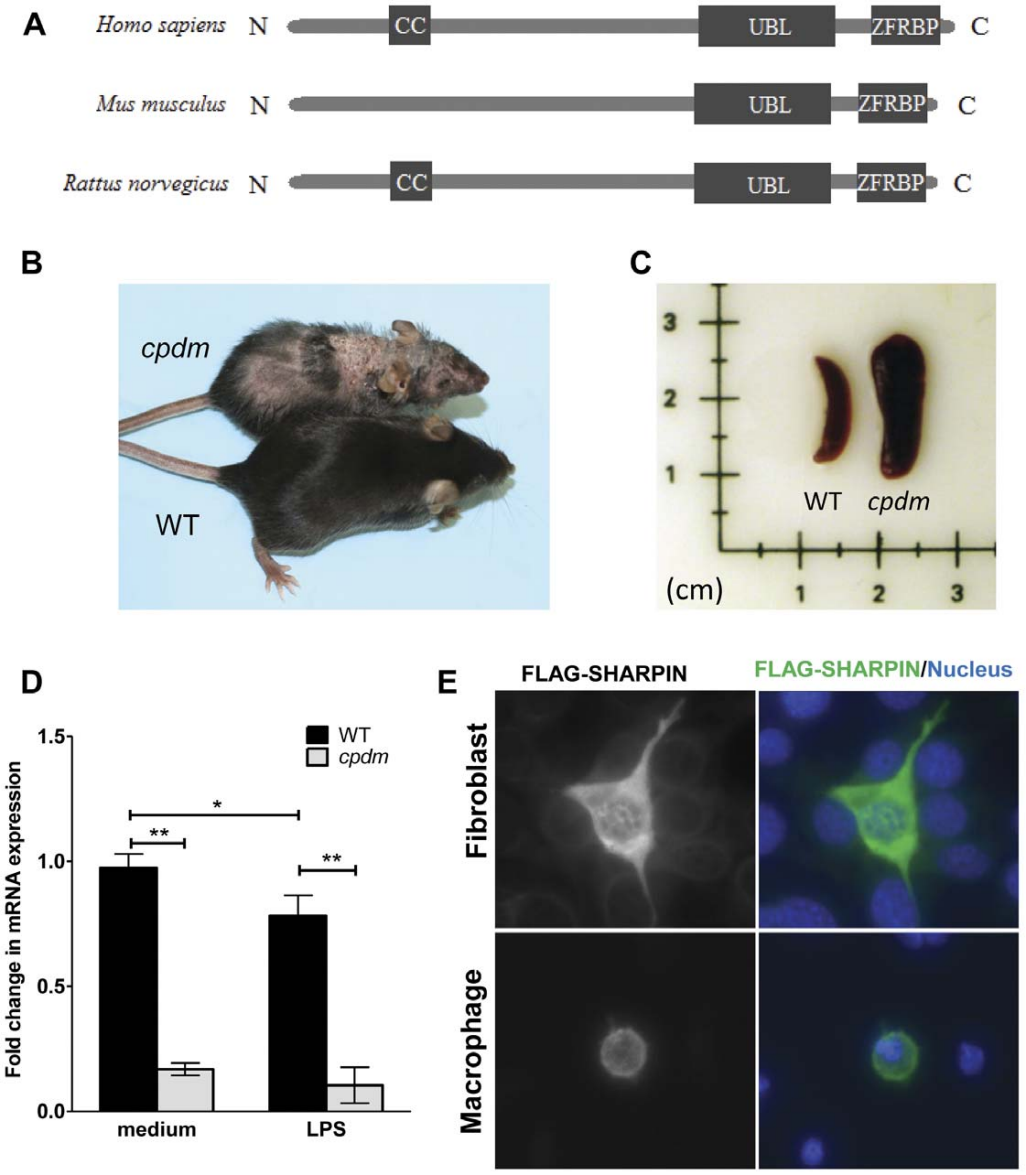
*In vivo* and *in vitro* features of SHARPIN. (A) COILS and MotifScan programs were used to predict the presence of CC (coiled-coil) domain, UBL (ubiquitin-like) domain and ZFRBP (zinc-finger Ran-Binding protein 2) domain which form similar motif patterns in the SHARPIN protein of human, mouse and rat origins. (B) Eight week-old females of WT and *cpdm* mice. The mutant mice (above) develop progressive skin inflammation starting at about four weeks. (C) Extramedullary hematopoiesis causes marked enlargement of the spleen of *cpdm* mice. (D) BMDC were incubated in the absence or presence of 100 ng/ml LPS for 4 hours. The mRNA level of *Sharpin* was measured by qRT-PCR and presented relative to the mRNA expression in non-stimulated WT BMDC. Bars represent the mean ± s.d. of 3 mice. * P<0.05; ** P<0.001. (E) Fibroblast (NIH3T3) and macrophages (RAW264.7) cells were transfected with the expression plasmid pFLAG-SHARPIN. After 48 hours, cells were fixed and probed with anti-FLAG and FITC-conjugated secondary antibody. Nuclei were stained with DAPI. In both transfected cell lines, FLAG-SHARPIN was found to be cytoplasm-localized.

Orthologs of SHARPIN protein are found in various species, including human, mouse and rat. Motif prediction programming, using COILS [11] and MotifScan [12], suggests that SHARPIN contains a coiled-coil (CC) domain, a ubiquitin-like (UBL) domain, and a zinc-finger Ran-binding protein 2 (ZFRBP) domain. These functional motifs constitute similar domain profiles that are present in the SHARPIN protein of all three origins (Fig. 1A), suggesting that SHARPIN exerts highly conserved functions across species. Spontaneous mutations in the mouse *Sharpin* gene results in a complex inflammatory phenotype characterized by severe dermatitis (Fig. 1B), systemic inflammation and an enlarged spleen (Fig. 1C) caused by extramedullary hematopoiesis [3]. The endogenous expression of *Sharpin* mRNA in BMDC was determined by quantitative real time-PCR (qRT-PCR) following culture in medium only or after stimulation with LPS. *Sharpin* mRNA was present in BMDC generated from WT mice (Fig. 1D) and its level was modestly decreased by LPS stimulation. There was a significant reduction of *Sharpin* mRNA (6–7-fold) in BMDC generated from *cpdm* mice. Transfection of *Flag*-tagged *Sharpin* in fibroblasts (NIH3T3) and macrophages (RAW264.7) indicated cytoplasmic localization of the SHARPIN protein (Fig. 1E).

### Phenotyping splenic DC and BMDC from WT and *cpdm* mice

**Figure 2:**
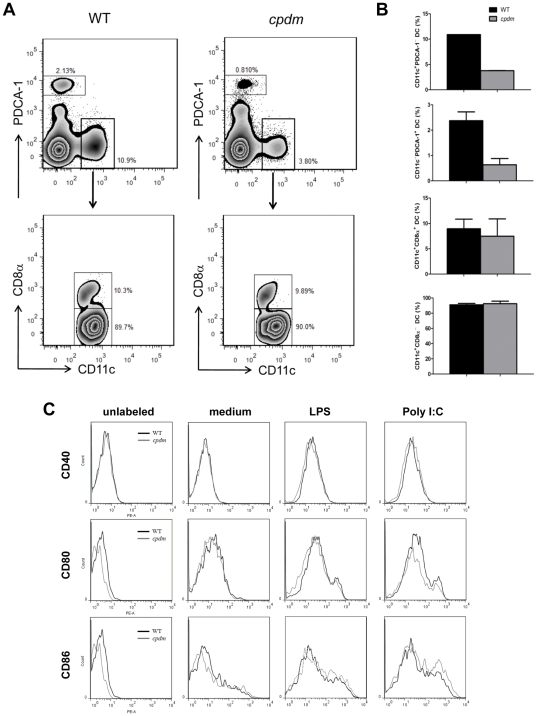
Effect of *Sharpin* mutation on DC subpopulations and maturation. (A) Spleens from WT and *cpdm* mice were isolated and subject to collagenase and DNase digestion. The obtained splenic homogenates were centrifuged over a Percoll gradient (35% and 55% density) for 15 minutes. The bands at the 35%-medium and the 35–55% interface were pooled, washed and stained with a combination of various antibodies to stain different DC subsets, conventional CD11c^+^CD8α^+^, CD11c^+^CD8α^−^ and plasmacytoid DC (CD11c^−^PDCA-1^+^).The top panels were gated on FSC^hi^SSC^lo^ cells to show separate populations of CD11c^+^PDCA-1^−^ and CD11c^−^PDCA-1^+^ cells. Further gating on the CD11c^+^PDCA-1^−^ subpopulation gave the bottom panel that showed two distinct pools of CD11c^+^CD8α^+^ and CD11c^+^CD8α^−^ cells. Percentages were calculated based on the parental population and were additionally shown as bar graphs (n = 2) (B). (C) WT and *cpdm* BMDC (5×10^5^) cells were stimulated with medium, 100 ng/ml LPS or 25 µg/ml poly I:C for 24 hours. The cells were labeled with PE-labeled anti-CD40, anti-CD80, and anti-CD86, and subjected to flow cytometry analysis. The populations shown in histograms were gated on CD11c^+^ cells. Unstained cells served as negative controls. Results are representative of two independent experiments.

DC are heterogeneous and can be categorized into multiple subtypes based on surface markers [13]. To determine if the *Sharpin* mutation affects DC development in lymphoid tissues, mouse spleens were examined for the distribution of conventional DC (cDC; CD11c^+^CD8α^+^ and CD11c^+^CD8α^−^) [13] and plasmacytoid DC (pDC; CD11c^−^PDCA-1^+^) [14]. The percentages for splenic cDC and pDC were both reduced in *cpdm* mice when compared with WT controls (Fig. 2A). However, when gated on CD11c^+^ cDC, the percentages of CD8α^+^ and CD8α^−^ cells were not affected by SHARPIN deficiency (Fig. 2A). Since the spleen of *cpdm* mice is markedly enlarged and contains three times as many cells (Fig. 1C), the different percentages of splenic cDC and pDC between WT and mutant mice reflect the increased number of total spleen cells rather than a reduction in cDC and pDC numbers. These data indicate that the *Sharpin* mutation does not affect the distribution of the examined DC subsets in the spleen.

BMDC from *in vitro* cultures functionally resemble non-lymphoid tissue DC and monocyte-derived inflammatory DC [15], [16]. The yields of BMDC from WT and *cpdm* mice were similar. BMDC were CD11c^+^ and MHC II^+^ with low expression of co-stimulatory molecules CD40, CD80, and CD86. The TLR3 ligand poly I:C and the TLR4 ligand LPS each activate overlapping but different signaling pathways and were used to induce DC maturation [17], [18]. Incubation with the TLR agonists for 24 hours resulted in increased expression of CD40, CD80, and CD86 on BMDC; however, there was no difference in the expression levels of these markers between WT and *cpdm* BMDC (Fig. 2B). Thus, SHARPIN deficiency did not influence the expression of co-stimulatory molecules by BMDC.

### Production of proinflammatory mediators by *cpdm* BMDC is impaired

**Figure 3:**
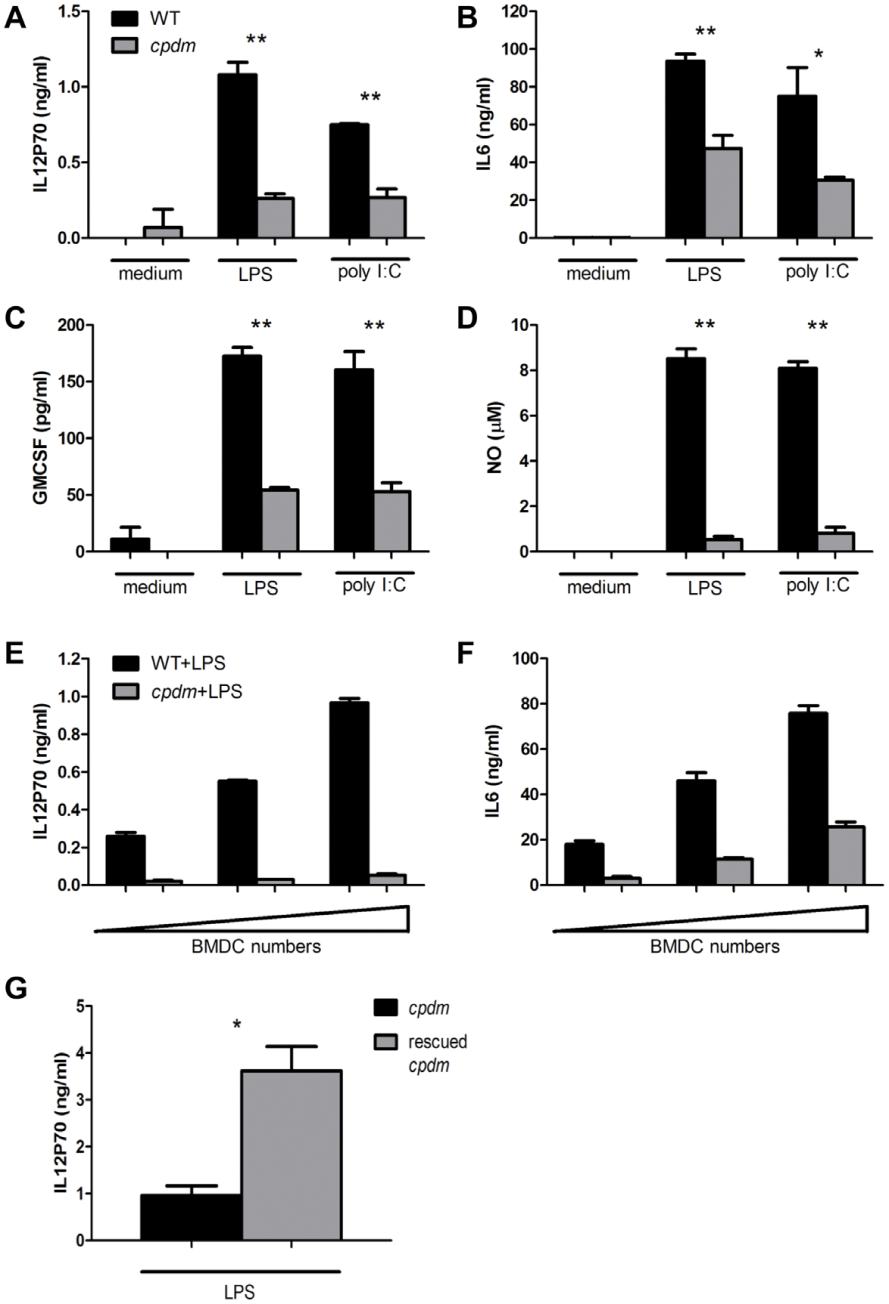
Defective production of pro-inflammatory mediators from stimulated *cpdm* BMDC. (A–D) Cultured WT and *cpdm* BMDC (1×10^5^ cells in 0.1 ml complete medium) were washed and stimulated with medium, 100 ng/ml LPS or 25 µg/ml poly I:C for 24 hours. Supernatants were collected for ELISA of IL12P70, IL6, and GMCSF, and for quantification of nitric oxide (NO). (E–F) Gradient numbers (1×10^4^, 2×10^4^, and 4×10^4^ cells in 0.1 mL complete medium) of BMDC were used for100 ng/mL LPS stimulation. After 24 hours, the amounts of IL12P70 and IL6 from the supernatant were measured. (G) The *cpdm* mice rescued by *Sharpin*-containing BAC had complete remission of the inflammatory phenotype [2]. BMDC developed from *cpdm* and rescued *cpdm* mice were plated (1×10^6^ cells in 0.3 mL) and stimulated with 100 ng/mL LPS. After 24 hours, supernatants were collected for analysis of IL12P70 production. Data are representative of three independent experiments. * P<0.01; ** P<0.005.

**Figure 4:**
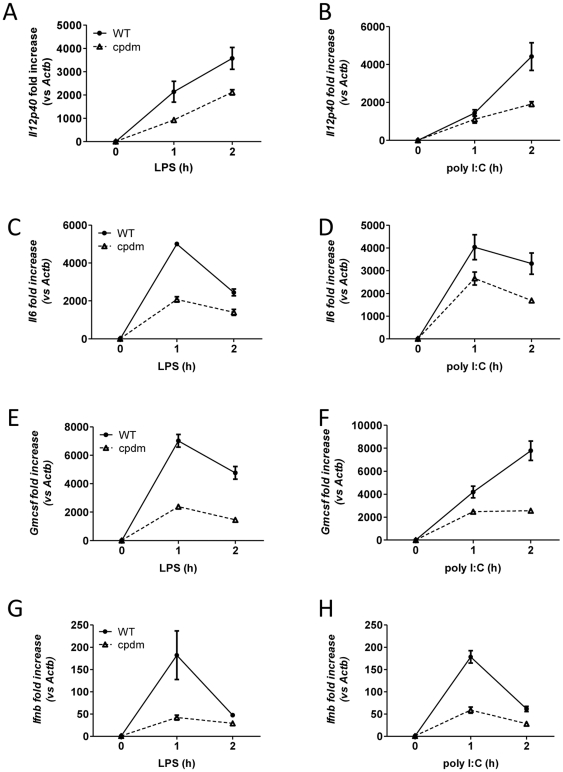
Decreased mRNA levels of inflammatory cytokines from *cpdm* BMDC. Cultured WT and *cpdm* BMDC (5×10^5^ cells in 0.2 ml complete medium) were washed and stimulated with 100 ng/ml LPS (A,C,E,G) or 25 µg/ml poly I:C (B,D,F,H). At 0, 1 and 2 hours, total RNA was extracted and subject to qRT-PCR to measure the expression of *Il12p40* (A,B), *Il6* (C,D), *Gmcsf* (E,F), and *Ifnb* (G,H) mRNA. Bars represent mean ± SD. Data are representative of two independent experiments.

Incubation with LPS or poly I:C resulted in secretion of IL6, IL12P70, and GMCSF from both WT and *cpdm* BMDC; however, BMDC from *cpdm* mice produced significantly less of all three cytokines compared with WT BMDC (Fig. 3A–C, E–F). The amount of nitric oxide generated by mutant BMDC was also significantly reduced compared with WT cells (Fig. 3D), indicating severely disrupted production of proinflammatory mediators from *Sharpin*-deficient BMDC. *In vivo* complementation with a *Sharpin* gene-containing BAC reversed the phenotype of the mutant mice [2], and BMDC generated from these rescued mice secreted significantly more IL12P70 than BMDC from *cpdm* mice (Fig. 3G), supporting a necessary role of SHARPIN for the production of IL12P70. In addition, the transcript levels of the inflammatory cytokines *Il6*, *Il12p40*, *Gmcsf*, and *Ifnb* were examined, and these were all significantly reduced in stimulated *cpdm* BMDC when compared with WT controls (Fig. 4).

### Impaired cytokine production by *cpdm* BMDC is correlated with selective defects in NF-κB signaling

There are a number of possible explanations for the defective cytokine secretion in stimulated *cpdm* BMDC, including 1) reduced surface expression of the LPS receptor complex, 2) increased production of anti-inflammatory mediators, 3) increased expression of negative regulators of TLR pathways, and 4) impaired TLR-induced signaling activation.

**Figure 5:**
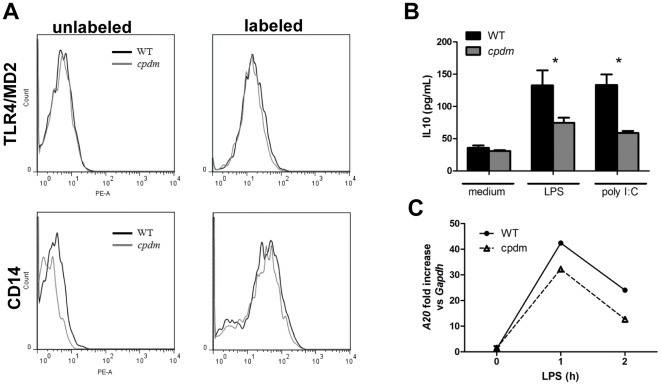
Normal TLR4 and MD2 expression and decreased IL10 secretion and A20 expression by *cpdm* BMDC. (A) Unstimulated WT and *cpdm* BMDC (5×10^5^) were labeled with PE-labeled anti-TLR4/MD2 or anti-CD14 and then subject to flow cytometry analysis. Unstained cells serve as negative controls. (B) Cultured WT and *cpdm* BMDC (1×10^5^ cells in 0.1 ml complete medium) were washed and stimulated with medium, 100 ng/ml LPS or 25 µg/ml poly I:C for 24 hours. Supernatants were collected for ELISA of IL10. (C) Cultured WT and *cpdm* BMDC (5×10^5^ cells in 0.2 ml complete medium) were washed and stimulated with 100 ng/ml LPS or 25 µg/ml poly I:C. At 0, 1, and 2 hours, total RNA was extracted and subject to qRT-PCR to measure the production of *A20.*

We determined the surface expression of the LPS receptor complex that comprises TLR4, the accessory proteins CD14 and myeloid differentiation factor 2 (MD2/LY96) [19]. Flow cytometric analysis shows that the expression levels of CD14 and TLR4/MD2 between WT and *cpdm* BMDC were similar (Fig. 5A). We then quantified the secretion of the suppressive cytokines IL10 that can inhibit IL12 secretion in an autocrine manner [20], [21]. The supernatants from LPS-stimulated *cpdm* BMDC contained significantly lower levels of IL10 than stimulated WT BMDC (Fig. 5B), suggesting that IL10 was not responsible for decreased secretion of IL12P70 by *cpdm* BMDC. Increased expression of a negative regulator of TLR signaling such as A20 [22] may also suppress cytokine secretion. However, the transcript level of *A20* was lower in LPS-activated *cpdm* BMDC than WT controls (Fig. 5C), thereby ruling out overexpression of A20 as a factor in the reduced cytokine production.

**Figure 6:**
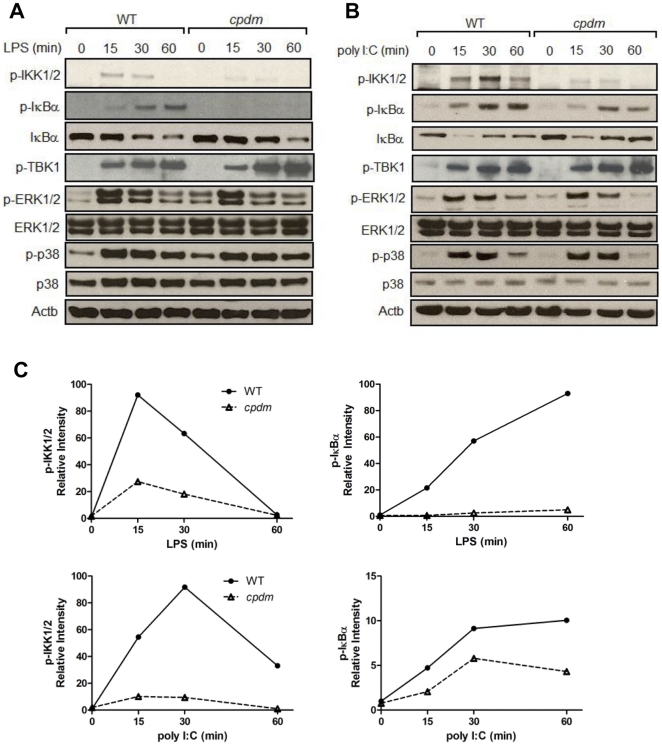
Inhibition of NF-κB signaling in *cpdm* BMDC. WT and *cpdm* BMDC (2×10^6^ cells in 0.5 mL complete medium) were stimulated with 100 ng/mL LPS (A) or 25 µg/mL poly I:C (B). At 0, 15, 30, and 60 minutes, whole-cell lysates were obtained and subject to immunoblots with antibodies against proteins involved in NF-κB, TBK1/IRF3, ERK1/2, and p38 signaling pathways. Beta-actin was used as loading control. (C) Cellular levels of p-IKK1/2 and p-IκBα in LPS- or poly I:C-stimulated BMDC were quantitated with ImageJ (NIH) and presented as trend lines. Results are representative of at least two independent experiments.

The transcription of TLR3/4-induced proinflammatory intermediates is tightly regulated by cellular signaling pathways, in particular NF-κB, TBK1/IRF3, and MAPK [23]–[27]. We next determined if disrupted NF-κB, TBK1/IRF3, and/or MAPK signaling may underlie the impaired cytokine production from stimulated *Sharpin*-deficient BMDC. Stimulus-induced phosphorylation of the IκB kinase (IKK1/2) is an essential step in NF-κB signaling, allowing phosphorylation and proteasome-mediated degradation of the NF-κB inhibitor IκBα to release the NF-κB transcription factors into the nucleus. The amount of phosphorylated IKK1/2 (p-IKK1/2) and IκBα (p-IκBα) following incubation with LPS or poly I:C was severely decreased in *cpdm* BMDC as compared with WT controls (Fig. 6). The *cpdm* BMDC exhibited similar levels of TBK1, ERK1/2, and p38 phosphorylation to those of WT cells (Fig. 6). These results indicate that the absence of functional SHARPIN decreased NF-κB activation but did not affect TBK1/IRF3, ERK1/2, and p38 signaling in BMDC.

### Th2-biased immunogenicity of stimulated *cpdm* BMDC

**Figure 7:**
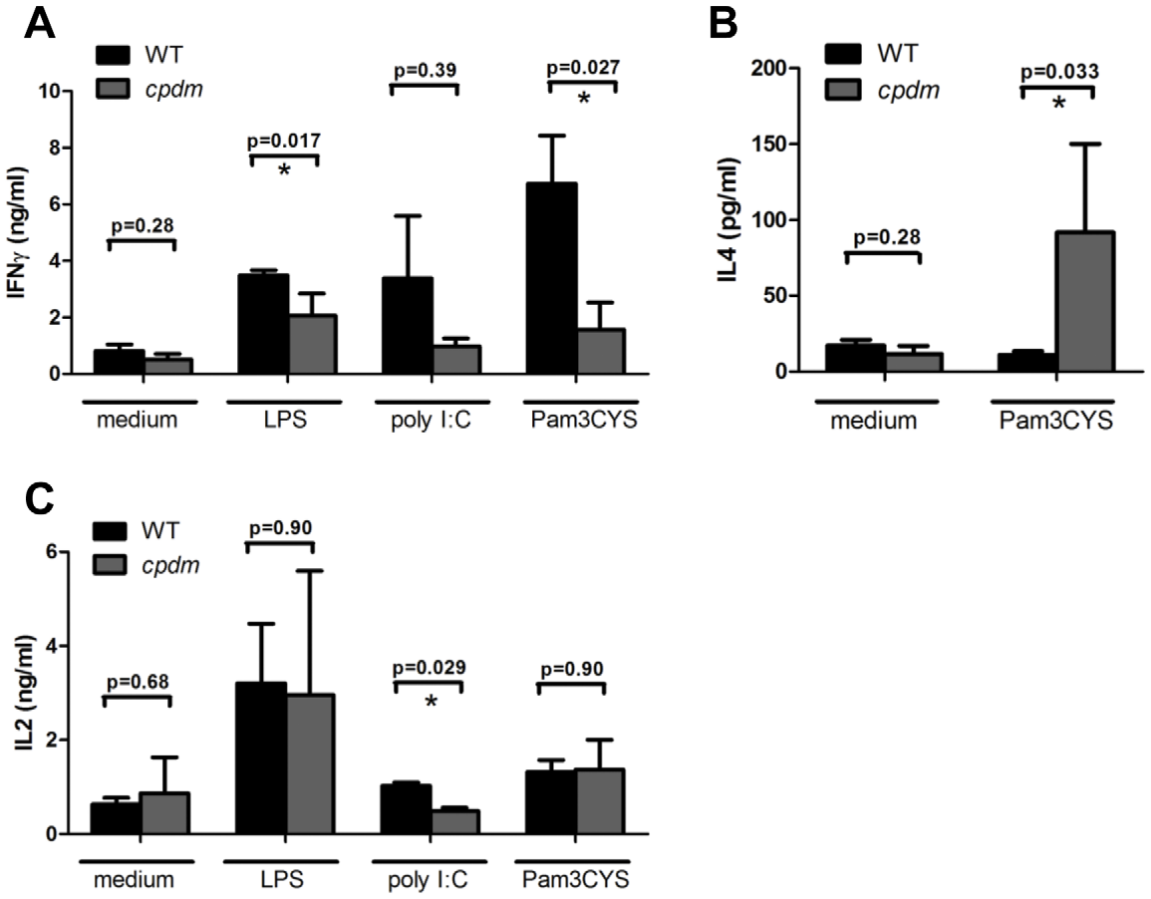
Stimulated *cpdm* BMDC induced Th2-biased cytokine production from naïve CD4^+^ T cells. WT and *cpdm* BMDC (5×10^4^ cells in 0.1 mL complete medium; MHC haplotype: H-2^b^) were incubated with medium, 100 ng/mL LPS, 25 µg/mL poly I:C or 5 µg/mL Pam3CYS. After 24 hours, cells were washed with PBS and incubated with freshly isolated allogeneic naïve CD4^+^ T cells (2.5×10^5^ cells in 0.1 mL complete medium; MHC haplotype: H-2^d^). After 5 days, supernatants were collected and the secretion of IFNγ, IL4, and IL2 were measured by ELISA. Negative controls are 1) stimulated BMDC without co-culture with allogeneic CD4^+^ T cells; 2) allogeneic CD4^+^ T cells without co-culture with stimulated BMDC. Samples from both negative controls had no detectable production of the aforementioned cytokines (not shown). Results are analyzed based on 2–4 mice per group. * P<0.05.

The defective IL12 production (Fig. 3A) and Th2-dominant cytokine profile in *cpdm* mice [5] suggest that the absence of SHARPIN affects the ability of *cpdm* BMDC to induce T cell differentiation into effector cells. Co-culture of allogeneic naïve CD4^+^ T cells with WT BMDC stimulated with LPS or poly I:C elicited robust IFNγ production, whereas the concentration of IFNγ in *cpdm* BMDC-T cell cultures was significantly lower after LPS stimulation (Fig. 7A), indicating impaired Th1-polarizing abilities of *cpdm* BMDC. In addition to TLR3/4 agonists, the TLR2 ligand Pam3CYS was used since it has been shown to induce both Th1 and Th2 responses [28]–[30]. Pam3CYS-matured WT BMDC induced robust IFNγ production at a significantly higher level than *cpdm* BMDC (Fig. 7A). The reduced Th1 differentiation following Pam3CYS stimulation is consistent with the recent report of decreased IL12 production in *cpdm* macrophages following TLR2 stimulation [31]. In contrast, more Th2-specific IL4 cytokine was produced in the *cpdm* BMDC co-cultures than the WT control (Fig. 7B), suggesting Th2-skewed immunogenicity of *cpdm* BMDC. The production of Th17-specific cytokine IL17A following LPS stimulation of dendritic cells was similar between stimulated WT and *cpdm* BMDC cocultures (data not shown). Despite the distinct Th1- and Th2-stimulating abilities of WT and *cpdm* BMDC, they were equally effective in IL2 production from BMDC-T cell co-cultures except for poly I:C stimulation where WT BMDC induced more IL2 than *cpdm* BMDC (Fig. 7C). The Th2-biased stimulating capability of *cpdm* BMDC when co-cultured with allogeneic naïve CD4^+^ T cells is consistent with the Th2-dominant cytokine phenotype observed in *cpdm* mice.

## Discussion

The present report showed that null mutations of the mouse *Sharpin* gene did not affect the steady-state distribution of splenic DC subsets nor the development and phenotype of BMDC. However, loss of SHARPIN significantly diminished the capacity of BMDC to secrete inflammatory cytokines and nitric oxide. The attenuated cytokine production was not due to the presence of anti-inflammatory inhibitors, and can be largely explained by selective inactivation of NF-κB signaling. Stimulated *cpdm* BMDC exhibited Th2-biased T cell-polarizing capabilities, consistent with the Th2 cytokine-dominant phenotype in *cpdm* mice. Together, these results indicate an indispensible role of SHARPIN in regulating DC immunological functions, disruption of which may contribute to the development of immune diseases.

Since WT and *cpdm* mice are both specific pathogen-free, the nature of the trigger of the severe inflammation in *cpdm* mice is not obvious. One such initiating factor could be endogenous apoptotic and/or necrotic cells that can release danger-associated molecular patterns (DAMPs) to launch and amplify an inflammatory response [32]. Such ‘sterile’ inflammation could be initiated and take place in all organs and tissues affected in *cpdm* mice, thus causing multi-organ inflammatory disorders. This hypothesis is supported by several recent studies. Fibroblasts of *cpdm* mice are highly sensitive to TNFα-induced cell death and the *cpdm* phenotype can be partially rescued by deletion of TNF [7], [8], suggesting that deficiency of SHARPIN compromises the anti-apoptotic mechanisms in *cpdm* mice resulting in cell death-induced inflammatory disease. Apoptosis of keratinocytes is a prominent feature of the skin lesions in *cpdm* mice [33] and this is mediated by caspase-dependent mitochondrial pathways [34]. These induced and/or intrinsic apoptotic cells can release various types of DAMPs that exert their pro-inflammatory properties by activating DC through pattern recognition receptors [32], [35], such as HMGB1 recognized by TLR2/4 [36], [37].

Consistent with the impaired Th1 immune response in *cpdm* mice [5], stimulated BMDC weakly polarized Th1 differentiation, but strongly supported the development of Th2 effector cells. Combined with the dramatic effect of IL12 treatment on the phenotype of these mice [5], this suggests that the Th2-biased systemic inflammation in *cpdm* mice is caused by reduced IL12P70 production from DC. The importance of IL12P70 in regulating Th1 and Th2 immune responses in mice was clearly demonstrated in IL12P35- and IL12P40-deficient mice [38], [39] and through clinical studies in human patients [40]–[42].

NF-κB, TBK/IRF3, and MAPK signaling are important pathways activated by LPS or poly I:C and disruption of either pathway may lead to decreased cytokine expression. NF-κB activation was selectively inhibited in LPS and poly I:C stimulated *cpdm* BMDC compared to wild type, while activation of TBK1/IRF3, ERK1/2, or p38 was not, indicating that disrupted NF-κB signaling in *cpdm* DC is responsible for the defective cytokine expression. These results seem to contradict a recent study that found increased levels of NF-κB activation and IL1 transcription in *cpdm* mice [43]. This difference can probably be attributed to the different cells and tissues used in these studies, and may point to cell- and tissue- type specific functions of SHARPIN. This is consistent with recent reports of tissue-specific effects of NF-κB signaling [44]. Ubiquitous activation of NF-κB by removing inhibitors such as A20 and ITCH through genetic manipulation results in widespread inflammation, consistent with the role of NF-κB in the production of pro-inflammatory mediators [45], [46]. However, selective inhibition of NF-κB activation in parenchymal cells of the skin, liver, and intestine results in chronic inflammation driven by NF-κB competent leukocytes [47]–[50]. This indicates that a balance between the pro-inflammatory role of NF-κB in leukocytes and the anti-inflammatory role in parenchymal cells is critical in the maintenance of tissue homeostasis. Experiments with mice with cell- and tissue-specific deletion of *Sharpin*, currently under way, will help to elucidate the role of SHARPIN in inflammation.

Despite defective NF-κB activation in the absence of *Sharpin* expression, there was no significant change in splenic DC populations or expression of co-stimulatory molecules on BMDC. Different NF-κB subunits involved in the canonical and non-canonical branches of the NF-κB signaling pathway have distinct functions to control specific aspects of DC development and function [51], [52]. The non-canonical pathway (p100 processing to produce p52) appears to be intact in SHARPIN-deficient cells [7], [9] suggesting that the NF-κB heterodimer p52/RELB is sufficient to maintain the normal regulation of DC homeostasis and maturation.

The molecular basis by which the *Sharpin* mutation causes reduced NF-κB activation in BMDC remains to be determined. LPS and poly I:C used here are well-defined ligands that specifically engage TLR4 and TLR3, respectively. The expression profile of surface TLR4 complexes is similar between WT and *cpdm* BMDC suggesting that defective NF-κB activation is not a result of differential TLR expression on target cells. LPS engages TLR4 to activate MYD88-dependent and TRIF-dependent pathways, whereas TLR3 stimulated by poly I:C only triggers TRIF-dependent signaling [53]. The defective NF-κB activation by both stimuli suggests that the *Sharpin* mutation interferes with the protein adaptors or kinases shared by both signaling pathways, such as RIP1 and TRAF6 [18]. Recent studies demonstrated that SHARPIN interacts with HOIP to form LUBAC that exerts its linear-ubiquitin-chain-ligase activity on NF-κB signaling players RIP1 and NEMO [7], an essential step for intact TNFα-stimulated NF-κB activation. The *Sharpin* null mutation disrupts the ubiquitylation process and abrogates the TNFα-induced NF-κB signaling pathway. Since TNFR and TLR partially share their downstream signaling cascades, a similar ubiquitin-mediated regulation may hold true for SHARPIN in LPS- and poly I:C-induced NF-κB activation.

In summary, the present study identified an indispensible role of SHARPIN in the production of pro-inflammatory mediators and TLR-induced NF-κB signaling. The impaired Th1-stimulating ability of *Sharpin*-deficient DC may account for the Th2-dominant inflammatory phenotype of *cpdm* mice. The balance between Th1 and Th2 differentiation is critical for immune homeostasis. A better understanding of how such balance is maintained will help design cytokine treatment for human diseases with Th2-biased cytokine secretion similar to the mouse *cpdm*, such as allergies and hypereosinophilic syndromes.

## Materials and Methods

### Ethics Statement

All mouse work was carried out in strict accordance with protocols approved by the Institutional Animal Care and Use Committees.

### Mice

Specific-pathogen free colonies of C57BL/KaLawRij-*Sharpin^cpdm^*/RijSunJ (JR#7599) and WT mice were obtained from The Jackson Laboratory (Bar Harbor, ME) and were maintained in a barrier facility. Normal littermate controls were either +/+ or +/*Sharpin^cpdm^*. These control animals were phenotypically indistinguishable and are referred to as WT. Sex-matched WT and mutant mice were used at 6–10 weeks of age. For some experiments, *cpdm* mice were crossed with transgenic mice with a bacterial artificial chromosome (BAC) containing the *Sharpin* gene (FVB/NJ-Tg(RP24-173I23)1Sun/Sun, JR#8279). These mice were backcrossed onto the C57BL/KaLawRij-*Sharpin^cpdm^*/RijSunJ background and N4 mice were used in the experiments reported here. All mouse work was carried out in strict accordance with the approved protocols by the Institutional Animal Care and Use Committee.

### Constructs and transfection

The complementary DNA (cDNA) of the mouse *Sharpin* gene was cloned and amplified from RAW264.7 RNA extracts. The primer sequences were forward, 5′-CC ATG GCG ATG TCG CCG CCC GCC GGC GGT; reverse, 5′- AAG CTT CTA GGT GGA AGC TGC AGC AAG A. The *Sharpin* cDNA was cloned into expression vector pFLAG-CMV-2. Murine fibroblasts and macrophages were were used to express recombinant SHARPIN protein. Cells (2×10^4^) were seeded in 96-well treated plates the day before transfection. After overnight incubation, 200 ng pFLAG-SHARPIN plasmids were transfected with 0.5 ul Lipofectamine 2000. 24 hours later, cells were incubated with fresh culture medium. After another 24 hours, cells were lysed to confirm the FLAG-SHARPIN expression by immunoblots with anti-FLAG. Cells with no transfection and transfected with empty vector pFLAG-CMV-2 were used as negative control in all transfection experiments.

### Generation of BMDC

BMDC were developed as previously reported [54], [55]. Cells were collected after 10–12 days of culture. The cell yield was 2–3×10^7^ cells/mouse with 80–95% BMDC.

### Phenotype of BMDC and splenic DC subsets

The phenotype of BMDC was determined before and after 24 hour culture in the presence of 100 ng/mL LPS. The cells were incubated in PBS with 0.1% NaN_3_, 1% BSA and 10% normal rabbit serum for 20 minutes on ice, washed and incubated for 30 minutes on ice with Alex Fluor-labeled anti-CD11c (MCD11C20, CALTAG) in combination with PE- anti-CD40 (3/23, BD Biosciences), PE-anti-CD80 (16-10A1, eBioscience), PE-anti-CD86 (P03.1, eBioscience), PE-anti-CD14 (Sa2.8, eBioscience) and biotinylated anti-TLR4 (BioLegend). The cells incubated with biotinylated anti-TLR4/MD-2 were washed twice and incubated for 30 minutes on ice with avidin-PE. To isolate splenic DC, spleens were collagenase digested and subject to Percoll gradient centrifugation. The bands at the 35–55% interface were collected to stain with PE-anti-CD11c (HL3, BioLegend), APC-anti-PDCA-1 (927, BioLegend) and FITC-anti-CD8α (53–6.7, BD BioScience). The cells were washed twice, fixed in 2% paraformaldehyde, and stored at 4°C until analysis. Flow cytometry was performed in an Excel (Coulter) instrument. Dead cells were omitted from the analysis by gating on forward and 90° light scatter, and 10,000 cells were analyzed by FlowJo software.

### BMDC-T cell *in vitro* interaction

WT and *cpdm* BMDC (5×10^4^) were stimulated with medium, 1 µg/mL LPS, 25 µg/mL and 5 µg/mL Pam3CYS. 24 hours later, cells were collected and washed with PBS. Allogeneic naïve CD4^+^ T cells were isolated from spleens of BALB/c mice by negative selection kit (Invitrogen) and were then added at 2.5×10^5^ and co-cultured with activated BMDC. After 5 days, supernatant was collected and the secretion of IFNγ, IL4, IL2, and IL17A measured by ELISA. Negative controls are 1) stimulated BMDC without co-culture with allogeneic CD4^+^ T cells; 2) allogeneic CD4^+^ T cells without co-culture with stimulated BMDC. Both negative controls show no production of aforementioned cytokines.

### RNA expression by BMDC

BMDC were cultured at 10^6^ cells/mL in 10 mL of RPMI-1640 complete medium in the presence or absence of 100 ng/mL LPS or 25 µg/mL poly I:C. After 1 and 2 hours, RNA was isolated with TRI-reagent (Sigma) according to the manufacturer's instructions. The expression of *Il6*, *Il12p40*, *Gmcsf*, and *Sharpin* mRNA was determined by qRT-PCR. Primers and probes were purchased from Applied Biosystems. Reverse transcription was performed at 42°C for 60 minutes with the final denaturation step at 90°C for 5 minutes in 30 µl containing 0.5 µg of total RNA. dNTPs, oligo(dT)_15_ primer, recombinant RNasin Ribonuclease Inhibitor, and M-MLV Reverse Transcriptase (all from Promega, Madison, WI) were used according to the manufacturer's instruction. Reverse transcription was done in a PTC-200 Peltier Thermal Cycler (MJ Research, Watertown, MA). qRT-PCR was performed in ABI Prism 7700 Sequence Detection System with TaqMan® Gene Expression Assays (Applied Biosystems, Foster City, CA) for mouse *Actb*, *IL6*, *IL12p40*, *Ifnβ*, *Gmcsf*, and *Sharpin* according to the manufacturer's protocol. The endogenous standard for normalization of the target gene was β-actin. Relative gene expression was calculated using the 2^−ΔΔCt^ method [56].

### *In vitro* cytokine secretion by BMDC

The BMDC were cultured in triplicate wells of 24-well or 96-well plates at 10^6^ or 10^5^ cells/mL, respectively, in RPMI-1640 complete medium. The cells were washed and stimulated with 100 ng/mL LPS or 25 µg/mL poly I:C. After 24 hours, supernatants were harvested for ELISA analysis. The presence of nitrite in the supernatants was determined using the Griess reagent.

### Immunofluorescence

Fibroblast and macrophage cells were transfected with pFLAG-SHARPIN. 48 hours later, cells were washed briefly with PBS, then fixed in cold methanol at −20°C for 10 minutes and cold acetone at -20°C for 1 minute. Incubate cells with PBS+1% BSA for 15 minutes to block non-specific binding. Fixed cells were then incubated with anti-FLAG (1∶100) as the primary antibody at room temperature for 1 hour, followed by three 5-minute washes. Cells were further incubated with goat anti-rabbit IgG-FITC (1∶100) as the secondary antibody at room temperature for 30 minutes, followed by three 5-minute washes. Cells were then examined using inverse fluorescence microscope.

### Immunoblots

After adding stimulating ligands, 100 ng/mL LPS or 25 µg/mL poly I:C, BMDC were collected and lysed at 0-, 15-, 30-, and 60-minute time points. Immunoblots were performed with antibodies (Cell Signaling Technology) against p-IKK1/2(#2697), p-IκBα (#9246), IκBα (#4814), p-TBK1(#5483), p-p38 (#9216), p38 (#9212), p-ERK1/2 (#4376), and ERK1/2 (#4695). Beta-actin (sc-47778, Santa Cruz Biotechnology) was used as loading control.

### Statistical analysis

Data are expressed as mean ± SD. The statistical significance of differences of means between experimental groups was determined by Students' t-test.
